# Perspectives of Mobile Versus Fixed Mammography in Santa Clara County, California: A Focus Group Study

**DOI:** 10.7759/cureus.494

**Published:** 2016-02-12

**Authors:** Yi-Ren Chen, Christine Chang-Halpenny, Narmadan A Kumarasamy, Angela Venegas, Clarence H Braddock III

**Affiliations:** 1 Department of Neurosurgery, Stanford University School of Medicine; 2 Radiation Oncology, Kaiser Permanente Southern California; 3 Radiology, Montefiore Medical Center; 4 Pediatrics, Montefiore Medical Cente; 5 Medicine, David Geffen School of Medicine at UCLA

**Keywords:** mobile mammography, prevention, women's health

## Abstract

Objective: Our aim was to examine underserved women’s perceptions on mobile versus fixed mammography in Santa Clara, California through a focus group study.

Background: Research has shown that medically underserved women have higher breast cancer mortality rates correlated with under-screening and a disproportional rate of late-stage diagnosis. The Community Health Partnership in Santa Clara County, California runs the Community Mammography Access Project (CMAP) that targets nearly 20,000 medically underserved women over the age of 40 in the county through the collaborative effort of an existing safety net of healthcare providers. However, little data exists on the advantages or disadvantages of mobile mammography units from the patient perspective.

Methods: We assessed underserved women’s perspectives on mammography services in Santa Clara County through two focus groups from women screened at mobile or fixed site programs. Patients were recruited from both CMAP clinics and a county hospital, and focus group data were analyzed using content analysis.

Results: We found that women from both the mobile and fixed sites shared similar motivating factors for getting a mammogram. Both groups recognized that screening was uncomfortable but necessary for good health and had positive feedback about their personal physicians. However, mobile participants, in particular, appreciated the atmosphere of mobile screening, reported shorter wait times, and remarked on the good communication from the clinic staff and empathetic treatment they received. However, mobile participants also expressed concern about the quality of films at mobile sites due to delayed initial reading of the films.

Conclusions: Mobile mammography offers a unique opportunity for women of underserved populations to access high satisfaction screenings, and it encourages a model similar to CMAP in other underserved areas. However, emphasis should be placed on providing a warm and welcoming environment for patients and ensuring the quality of mammography images.

## Introduction

Breast cancer is the most common cancer among women in the United States, with up to 52% of late-stage breast cancers detectable by a screening mammogram one to three years prior to diagnosis [1]. It is the number one cause of cancer death in Hispanic women, and the number two cause of cancer death in Caucasian, African American, Asian/Pacific Islander, and American Indian/Alaska Native women [2]. Research has shown that medically underserved women have higher breast cancer mortality rates correlating with underscreening and a disproportional rate of late-stage diagnosis [3]. One proposed intervention is the use of mobile mammography, which may improve screening rates by reducing barriers due to limited access.

To date, there are only a few studies looking at the efficacy of mobile mammography services. In 1992, 240 mobile mammography facilities were operational in the United States, all accredited by the American College of Radiology. In a survey of 1,057 screening facilities, only 2.4% were identified as mobile, with this method of screening accounting for 3% of total mammography exams performed*.* On average, the mobile facilities operated at one-third higher volume than stationary facilities. Mobile facilities have also been found to have more services during evening hours, more screening than diagnostic exams (70% vs. 50%, p < 0.05), and a higher proportion of women who were self-referred for screening (28% vs. 7%) [4]. Other studies have also shown that mobile services are more likely to have lower screening fees, which may allow for increased access [5]. However, little data exists on both the community response and women’s perception of mobile mammography.

Our study proposes to examine underserved women’s perceptions on mobile versus fixed mammography in Santa Clara, California through a focus group study. We partnered with the Community Health Partnership, Inc. (i.e. the Partnership), a consortium of non-profit community health center networks with 31 sites in Santa Clara and San Mateo counties. In particular, we worked with the Community Mammography Access Project (CMAP), a branch of the Partnership that uses low-cost mobile mammography to increase access to screening in an effort to reduce late-stage breast cancer diagnoses among uninsured and underinsured women in Santa Clara County. The prevalence of breast cancer in Santa Clara County reflects national trends as the most common cancer among women and the second most common cause of all cancer-related deaths after lung cancer. At the time of this study, there were nearly 20,000 low-income, medically underserved women over the age of 40 living in Santa Clara, California, with many facing significant barriers to care, such as limited access to transportation, delayed appointments at county safety-net hospitals, and language barriers. Despite efforts like those of the "Cancer Detection Program Every Woman Counts" to improve access, only 20% of the 36,000 eligible women in the Central Coast region utilize services and only 30% are re-screened one year after their initial screening [6]. Since CMAP first piloted its integrated model of mobile mammography at three community health center sites in 2006, it has screened over 4,000 women at 14 clinic sites in Santa Clara and San Mateo counties.

In our focus group, we assessed women’s general opinions and concerns related to mammography services from mobile sites at various community clinics or the fixed county hospital within Santa Clara County. We also focused on accessibility (location, transportation), comfort (atmosphere, interaction with staff members), communication of results, follow-up, and the advantages and disadvantages of mobile mammography sites versus fixed mammography sites. We hope that the results of our study will be helpful in generating further qualitative and quantitative studies on mammography services in underserved areas.

## Materials and methods

### Design

We chose to utilize the focus group methodology for our study, as a means to generate hypotheses and new information.

### Location

Our study took place in Santa Clara County, California. Of the 14 clinic sites that used mobile mammography, we recruited patients from the Indian Health Center, the San Jose Foothill Clinic, and the Asian Americans for Community Involvement Clinic. Fixed mammography participants were recruited from a county hospital (Santa Clara Valley Medical Center). 

### Recruitment

Mobile mammography participants were recruited from community clinics that partnered with CMAP between June and July 2008. Our target population was uninsured or underserved women (served by CMAP clinics) over the age of 40 who had received a mammography screening in Santa Clara, California within the past two years. Participants were also screened by phone for verification and as a reminder of the focus group event. Each participant received a $20 grocery store gift card to compensate them for their time.

### Focus groups 

Two focus groups were conducted: one with women who received mammograms from mobile sites and the other with women from the fixed site. The focus groups were hosted by the Community Health Partnership office, which is geographically close to both the mobile sites and fixed site. Informed consent was obtained from all participants. The moderator welcomed everyone, conducted introductions, and used a script to ensure coverage of key topics during the focus group. Both focus groups were conducted in the English language. The three main study questions were: 1) How easy was it to get a mammogram at the given site or mobile service? 2) How comfortable were women at the given site or mobile service, and how good was the follow-up? 3) What were their opinions on mobile versus fixed mammography, including advantages and disadvantages?

After the focus groups, the women were asked to complete a brief demographic exit survey with queries of the participant’s age, marital status, zip code, ethnicity, occupation, insurance status, household income, people with whom they were living, education, distance from their clinic (or fixed site) to home, location, and transportation to the clinic. We also noted the date and location of their last mammogram, specifically, whether it was at a mobile or fixed site. Lastly, we asked whether women would prefer to go to a mobile or fixed site for their mammography screening, and we inquired about their reasons and how strongly they felt about their preference. 

Both focus groups were audio-taped and transcribed. In addition to the moderator, the focus groups also had a designated note-taker sitting in the back of the room, who recorded themes and interpersonal interactions that may have been difficult to understand using only the audio tape. 

### Data analysis 

Audio-tapes were converted into transcripts for content analysis. Content analysis is a standard method in social science research for organizing the information gathered from a focus group or interview into a series of common themes or ideas expressed. All transcripts were checked for accuracy and words were recorded verbatim. The moderator, as well as the note-taker, independently checked the transcription. Finalized transcripts were not shared with focus group participants. 

The researchers developed a series of categories, or coding frames, that served as a framework to identify common themes, issues, or ideas expressed throughout the two focus groups. A systemic approach was utilized, beginning with open coding to look for differences and similarities in the data. To ensure intercoder reliability, each researcher individually categorized and coded the text into several broad themes based on attitudes, perceptions, and other issues. Based upon comment themes found in these separate reports, a condensed master coding frame was made to further analyze the data.

The Institutional Review Board at Stanford University Medical Center approved this study (approval #19126).

## Results

In total, 11 participants were recruited, representing several ethnicities (Asian, Latino, African-American, Caucasian, and Vietnamese). Seven participants were in the mobile mammography focus group, and four were in the fixed site focus group. Demographic information between the two focus groups varied slightly. Seventy-one percent of mobile participants self-identified as Latino, 14% as Caucasian, and 14% as Asian, whereas 25% of fixed group participants self-identified as Latino, 50% as Caucasian, and 25% as Asian. In addition, 71% of mobile participants cited Spanish as their primary language compared to 14% speaking English and 14% Vietnamese. Seventy-five percent cited English as their primary language and 25% cited Spanish in the fixed group. Six major themes were identified throughout each focus group session: motivation for screening, perceptions of clinic staff, comfort, wait time, communication and continuity of care, and impressions about mobile mammography.

### Motivation for screening

Women from both groups shared the same motivations for obtaining a mammogram. Several women in both groups had personal ties to friends and family with breast cancer. They also expressed a general interest in maintaining their health.

“I don’t like getting mammograms, but if it means my life [I will]. So it's something that has to be done that has to take care of cancer in my body.” (Mobile site user)

### Perceptions of clinic staff

Both groups expressed trust and positive feedback about personal physicians.

Fixed site participants focused on their lack of familiarity with the clinic staff and their strong preference for female technicians.

Mobile mammography participants praised the CMAP staff for being vigilant with scheduling and appointments, as well as treating them sympathetically.

“My doctor she tells me about the self-exam. She schedules me for the mammogram and she showed me how to do it [self-exam]. She showed me on my breast how I should roll it and she gave me pamphlets to read. And I have this little plastic card that I put up over my shower head so that when I take a shower, I feel [does the self-exam].” (Mobile site user)

### Comfort

Both groups recognized that, even though mammograms were uncomfortable, screening was necessary to detect cancer.

When asked about their comfort level, mobile participants tended to focus more on the atmosphere of the clinic (casual atmosphere, availability of snacks, pink decorations), whereas the fixed group focused more on the procedure itself.

“Can’t think of anything to change. I mean, it’s uncomfortable, but that’s what you got to do, that’s life. You’ve got to do what you’ve got to do.” (Fixed site user)

### Wait time

Both groups experienced long wait times.

The mobile group participants said that they were always informed about the reason for appointment delays.

“And you wait. And they [fixed site] always say the same thing. They say they are running behind. They are always running behind.” (Fixed site user)

### Communication and continuity of care

The mobile participants praised the scheduling practices of local clinics, mentioning that they received frequent calls and reminders before their scheduled mammogram.

The fixed site participants complained about poor communication from the medical staff at the county hospital.

“You are reading a letter that has this … your right breast contains this 1 cm by 1 cm lump at this time we don’t find that it is cancerous, see you in six months. I think that is kind of cold” (Fixed site user)

### Impressions about mobile mammography

Participants in the mobile group were concerned about the quality of images, citing that fixed sites usually had careful technicians who took multiple images, whereas mobile site technicians seemed less meticulous.

“It seemed to go a lot quicker than those times at [fixed site] where they spent a lot more time adjusting it and getting it just right so I was kind of curious about that…and I kind of mentioned it because that’s the kind of person I am, I said 'Are you sure you guys did this shot [view] there?' " (Mobile site user)

## Discussion

The results from our study suggest that overall women had a pleasant experience with mobile mammography and commended the frequent reminders before an appointment from the clinic staff. However, they had some reservations regarding the quality of the screening at the mobile sites compared to fixed sites.

A welcoming environment was established at the CMAP events through pink site décor and staff uniforms, as well as free snacks and drinks in the waiting area. Furthermore, the centers were arranged to allow those waiting to watch health promotion videos on the importance of self-breast exams and mammography screenings.

One randomized trial by Reuben, et al. has shown that offering health education and immediate access to mobile mammography at community clinics is particularly beneficial for older women and some subgroups who may traditionally have low screening rates [7]. Although many of the women in the focus groups were already motivated to be screened, the CMAP visits provided additional opportunities to promote further health screening (i.e., blood pressure checks, lipid studies, and colonoscopies) or to encourage positive lifestyle changes. As a community-based program, these screenings are also an opportunity to encourage women to take an active role in their healthcare, or even to promote health education or screening among their family and friends.

With regards to communication, focus group participants praised the mobile mammography screening sites for frequent calls and reminders for their appointments. However, patients in both groups were disgruntled with follow-up results appearing in letters rather than more personal discussion, but there was no difference between the two groups. In contrast, other studies have identified follow-up communication as one of the chief problems plaguing mobile mammography units. Of 636 patients screened for breast cancer via mobile units in the Cook County Bureau of Health Services in Illinois between 2001 to 2002, almost 80% did not have follow-up results documented [8]. In another study, women with abnormal results have cited being unaware of the results, misunderstanding the results letter, or considering the abnormal mammogram “unimportant” or “feeling fine.” This unfortunately resulted in untimely or inadequate follow-up of abnormal results. Some women also had concerns about the potential costs of follow-up and difficulty arranging appointments, while others cited competing illness, fear of further testing, or transportation barriers [9]. Some of these difficulties may have been circumvented if these women had a primary care provider or had regular checkups at their safety-net health centers.

Women in our study who had obtained mammograms at both fixed and mobile sites also expressed concern about a decreased quality of images at the mobile sites because of fewer images taken and technicians being less particular about the images they obtained. There are two issues at hand: the intrinsic quality of the mobile mammography unit and the capability to get additional images at the time of screening. Firstly, it is possible that the images from mobile sites are not as high quality as those at county hospitals simply due to the need for portable, light machines. Women may need to be reassured about the adequacy of the machines if they have had mammograms at other sites. Secondly, the initial processing and reading of mobile mammograms could not be done while the women were at the clinic. Thus, on occasion, women would need to return for additional views at a later date, potentially causing additional anxiety and inconvenience. As the technology for mobile units improves, for example, with digital imaging and instant reading of films, these disparities will hopefully decrease.

In addition, sustainability, funding, and patient eligibility are also issues to consider for mobile mammography services. In a 1996 survey of mobile units, 52% of mobile mammography units reported financial losses due to several logistical problems. These problems include downtime due to maintenance (affecting 77% of those surveyed), vehicle problems (71%), bad weather (65%), and equipment damage [5]. These issues suggest that it may be necessary to subsidize mobile mammography services in order to ensure quality and sustainability. CMAP is funded by a state program, but such funding availability varies state by state. Furthermore, public health officials should also be mindful of patient eligibility and insurance status. In assessing why women above 40 sought follow-up care outside the safety-net system, 40% of women in one study cited having insurance or a primary physician as the primary reason. Screening women with insurance through mobile sites would be a loss of limited resources because these women have fewer problems with access and can have mammograms through their insurance [8]. Currently, recommendations for mammography screening vary among different organizations: American Cancer Society [10], American College of Physicians [11], American College of Radiology, American Medical Association, National Cancer Institute, American College of Obstetrics and Gynecology [12], and U.S. Preventive Services Task Force [13]. In general, it is suggested that women aged 50 and above should have annual mammograms. No consensus has been reached for whether women aged 40-49 should be screened [13].

Some limitations of our study include the small number of participants and the fact that the focus groups were conducted in English. Additional thematic content may have arisen from including Spanish or Vietnamese-speaking women. As our study was meant to be hypothesis generating, further qualitative and quantitative studies focusing on the topics generated in our focus groups may be useful for further assessing the efficacy of mobile mammography services. 

We believe the results of our study not only commend the use of mobile mammography to reach underserved women, but also carry specific implications that may bolster the effectiveness of screening. For one, programs can use screening visits as an opportunity to not only teach women's health issues (i.e., self-breast exam, pap screening, and HPV vaccine for children), but also to promote general health screening (i.e., cholesterol check, blood pressure, and healthier cooking). If feasible, it would be helpful for women if social workers were on site to provide guidance on how to obtain medical services for those who cannot afford it. To ensure good, patient-centered communication, centers should provide interpreter services (also possible through the phone) and clear points of contact for those with queries regarding results or appointments. Lastly, women should be told ahead of time that they may be called for additional images due to the technology used at mobile sites. It may be a worthy investment to purchase machines with digital images that can help minimize the need for repeat follow-ups.

## Conclusions

There is currently a dearth of research justifying the higher cost of mobile mammography services. With the economic downturn and budget crisis, many mobile mammography services are in danger of losing funding. Our study aims to provide an objective pilot assessment of the efficacy of mobile services, specifically focusing on women's perspectives on convenience, comfort level, and follow-up. In general, mobile mammography participants enjoyed a more comfortable atmosphere and experienced better customer service and follow-up from staff members. They also reported shorter wait times compared to fixed site participants. However, there was concern about the quality of films at mobile sites due to a perceived lack of technician meticulousness and delayed initial reading of the films. Future studies with larger numbers of participants and more targeted quantitative data on accessibility, comfort, and follow-up can help policymakers and community health partners expand safety nets like the one in Santa Clara County, California to reach more underserved populations. 
